# Petroleum in Bronchitis

**Published:** 1881-05

**Authors:** 


PETROLEUM IN BRONCHITIS.—In a communication to the *Gazette des Hôpitaux*, Dr. Moube gives his experience of petroleum capsules in simple and chronic bronchitis. This balsamic had been brought before the Therapeutical society by Dr. Blache, a year ago, at the suggestion of a Paris chemist, who named it Gabian oil, in order to prevent public prejudice. Each capsule contains twenty-five centigrams of pure petroleum, the ordinary oil not being used, as it has to be distilled in contact with sulphuric acid to render it fit for lighting purposes. It is stated that at the Hôpital Beaujon, where these capsules have been freely ordered for chronic bronchitis, a rapid diminution of the secretion and of fits of coughing has been observed ; and in tuberculosis, also, the medicine has given encouraging results.

---

"MY husband is a brute," declared Mme. X. to an intimate friend the other day. "Why, my dear, what's the matter now ?" "He found fault with a little vivacity of mine yesterday, and I threw a candle-stick at his head ; then what do you suppose he did ?" "I don't know." "Why, he stood before the mirror, so that I couldn't throw the other. The brute !"

### WHY WE SHALL NEVER STARVE.

According to a recent compilation and comparison of the statistics of grain production of the country made by the Chicago *Times* the wheat yield of 1871 was 230,722,400 bushels, in 1875 it was 292,136,000 and in 1880 it was 480,840,723. In the same period the yield of corn increased from 991,898,000 bushels in 1871 to 1,537,535,940 bushels in 1880, the Western states producing the bulk of corn as well as wheat. The grain area of 1880 was 104,142,676 acres, a large total, but yet 70,000,000 less than the single State of Texas contains. The average yield per acre of wheat in 1880 was 13.3 bushels ; of corn 28.9 ; of oats, 27.8 ; of barley, 25.1. The value of the grain products of the United States since 1871 is put down at $10,000,000,000. The value of last year's crop is divided as follows : Wheat, $453,558,371 ; corn, $606,685,371 ; other grains, $177,389,269. The growth of the export movement of grain has been constant during the decade, except in 1875 and 1877. In the former year there was a decrease of 18,000,000 bushels and in the latter less than 1,000,000. In 1871 the total exports of grain from all ports of the United States were 72,122,398 bushels ; in 1880, 289,537,074 bushels.

### "STRAWBERRIES, RIPE STRAWBERRIES!"

"Strawberries ! Ripe strawberries!"
Shouted big Johnny Strong;
And he sold his baskets readily
To folks who came along.

But soon a tiny voice piped forth,
" Me, too !" Nell could not shout
As John did. Yet she too must sell
The fruit she bore about.

"Ho, straw-berr-e e-s!" roared lusty John,
" Me, too!" piped Nell, so sad.
And Johnny made good sales that day,
But Nell sold all she had.

—*St. Nicholas for June* 1881.

Land in England.—Behind the land question in England is the question of habits. The land is held in these great tracts, not for enjoyment, but from pride. The brains of men are so equal under modern facilities of education that hereditary descent would be inconsequential if it did not have hereditary estate to advertise the fact ; and consequently every parvenu in the nobility rushes to buy land at a ridiculous price, so that he can appear on the landscape of the country among the sons of Normans. For the greater part of the year an English estate is not fit to live on, on account of the climate, and there is plenty of land to be had for less than one dollar per acre on the steppes of the Rocky Mountains more agreeable for hunting, for residence, and for every other enjoyment, than the best land in England.

